# Comparison of deep learning models for real-time neural tissue segmentation in spinal endoscopy

**DOI:** 10.1186/s12880-025-01918-4

**Published:** 2025-11-17

**Authors:** Wounsuk Rhee, Hyung Rae Lee, Bong-Soon Chang, Sam Yeol Chang, Hyoungmin Kim

**Affiliations:** 1 Ministry of Health and Welfare, Government of the Republic of Korea, 13, Doum 4-ro, Sejong, 30113 Republic of Korea; 2 Siebel School of Computing and Data Science, University of Illinois Urbana-Champaign, 201 N. Goodwin Avenue, Urbana, IL 61801 USA; 3 Department of Orthopedic Surgery, Korea University Anam Hospital, 145, Anam-ro, Seongbuk-gu, Seoul, 02841 Republic of Korea; 4 Department of Orthopedic Surgery, Seoul National University College of Medicine, 103, Daehak-ro, Jongno-gu, Seoul, 03080 Republic of Korea; 5 Department of Orthopedic Surgery, Seoul National University Hospital, 101, Daehak-ro, Jongno-gu, Seoul, 03080 Republic of Korea; 6 Healthcare AI Institute, Seoul National University Hospital, 101, Daehak-ro, Jongno-gu, Seoul, 03080 Republic of Korea

**Keywords:** Endoscopic spine surgery, Neural tissue segmentation, Deep learning, Computer vision, Real-time image segmentation

## Abstract

**Background:**

In biportal endoscopic spine surgery (BESS), accurately identifying neural structures, mainly the spinal nerve roots, dural sac, and the cauda equina, is crucial for preventing dural tears and achieving optimal clinical outcomes. Contrary to the growing popularity of deep learning in biomedical image processing, its application to BESS has not yet been well established. We propose a two-stage framework for real-time neural tissue segmentation from spinal endoscopy video, and compare various types of deep learning architectures.

**Methods:**

6410 intraoperative images from 28 patients were collected and split at the patient level into 4661 and 1749 images for training and testing, respectively. For each set, 2307 and 635 images contained neural tissue. First, a lightweight image classifier that determines the presence of neural tissue was developed. Then, six variants of the U-Net family and SegFormers for neural tissue segmentation were trained. Ground truth segmentation masks were generated by a spine specialist with more than four years of experience. AUROC and DSC on the test set were the primary outcome measures for the classification and segmentation models, respectively, and computational burden was also measured, followed by qualitative assessment of the output predictions.

**Results:**

ResNet-18 achieved the highest test AUROC of 0.92 (95% CI: 0.91–0.93), running at a mean inference time of 1.2 ms per image, outperforming MobilenetV3-Large in all performance metrics (*p* < 0.001) and computational efficiency. AR2U-Net exhibited the highest test DSC of 0.80 (95% CI: 0.78–0.81), IoU of 0.70 (95% CI: 0.68–0.72), and AUPRC of 0.88 (95% CI: 0.87–0.89). The test performance metrics of other U-Net variants did not differ significantly, except for AUPRC (*p* < 0.001), while those of SegFormer-B0 and SegFormer-B1 were significantly inferior (*p* < 0.001). U-Net variants were generally operable at a reasonable speed (25–40 FPS), in which U-Net and AU-Net exhibited inference times of less than 30 ms.

**Conclusions:**

We have developed and compared deep learning models for neural tissue segmentation in spinal endoscopy and explored their potential for clinical application to real-time surgical video streams.

**Clinical trial number:**

Not applicable.

## Introduction

Biportal endoscopic spine surgery (BESS) is widely performed in patients requiring surgical decompression of the spinal canal [1, 2]. It is usually preferred over open spine surgery because of its advantages, such as smaller incisions, reduced postoperative pain, and shorter hospitalization periods [2, 3]. However, complications, including dural tears, nerve root injury, and epidural hematoma, may still occur in BESS, particularly when performed by less experienced surgeons in the early stages of the steep learning curve [4–6]. Among these complications, dural tears are the most common, occurring in approximately 4% [5]. They require substantial clinical attention as inadequate treatment may lead to headache, nausea, delayed recovery, or, in severe cases, neurologic deficit, infection, and the need for revision surgery. Therefore, accurately recognizing the neural tissue – spinal nerve roots, the dural sac surrounding the spinal cord, and the cauda equina – as well as promptly managing dural tears, is essential to ensuring optimal clinical outcomes [6].

Over the past decade, the rapid development of artificial intelligence (AI) has extended the frontier of biomedical research, leading to applications ranging from organ segmentation to cancer risk stratification and radiology-based diagnostics [7–11]. Endoscopic imaging has been an active area of research in computer vision; however, applications in spinal endoscopy remain limited compared to those in colonoscopy or laparoscopic surgery [12–15]. Cho et al. developed surgical instrument tip detection modules for BESS images with YOLOv2 and RetinaNet, while Lee et al. proposed a U-Net-based model for neural tissue segmentation in spinal endoscopic images [15, 16]. Deep learning models capable of simultaneously distinguishing various types of tissues and instruments have also been proposed [17].

While prior studies have demonstrated that deep learning models can achieve satisfactory performance in spinal endoscopy, the field is still in its early stages and presents several limitations. First, there is a lack of rigorous analysis regarding computational resources and inference speed, both of which are critical for real-time applications. In addition, the diversity of model architectures remains limited, and recent advances in computer vision, such as transformer-based models, have yet to be incorporated into this domain. Moreover, the handling of false predictions remains insufficient, limiting the clinical applicability of existing approaches.

In this study, we propose a two-stage model that first classifies the presence of neural tissues in BESS images and then performs segmentation, aiming to reduce false predictions. In addition, we thoroughly evaluate the models’ computational demand to assess the feasibility of their real-time application on modern hardware. Furthermore, to ensure architectural diversity in the segmentation module, six U-Net variants as well as SegFormer, a transformer-based segmentation model, are studied [18–24]. The following sections provide details on data acquisition, a brief theoretical background of architectural components used in our study, and specifications for model training. For each model, prediction performance, computational burden, and inference speed are reported to promote a comprehensive understanding of the results. Finally, we discuss the clinical applicability of the proposed models and suggest future directions for our research.

## Methods

### Data acquisition

Upon approval of the Public Institutional Review Board of the National Bioethics Policy Institute and obtaining a waiver of informed consent, video recordings of 29 BESS operations performed on 28 patients were retrospectively collected. One patient received two operations on separate dates, while others received one endoscopic spinal surgery each. Lumbar interlaminar decompression was the most common procedure, performed in 21 patients, followed by 5 cases of lumbar formainal decompression and 3 cases of cervical foraminotomies. The cohort was randomly split at the patient level into train-validation and test sets, each consisting of 22 and 6 patients, respectively, and their demographic characteristics are described in Table 1.

**Table 1:** Demographic characteristics of train-validation and test sets

	Train-validation set	Test set
Number of patients	22	6
Number of operations	22	7
Lumbar interlaminar decompression		
L2-3	1	0
L2-4	1	0
L3-4	1	0
L3-5	0	2
L4-5	13	2
L5-S1	0	1
Lumbar foraminal decompression		
L3	1	0
L4	1	1
L5	2	0
Cervical foraminotomy		
C4-7	0	1
C5-7	2	0
Age in years	65.4 ± 10.7	63.8 ± 14.1
Sex		
Male	9	3
Female	13	3
Number of images, total	4661	1749
Number of images, nerve root or spinal cord present	2307	635

The first row indicates the number of patients with the number of obtained images in parentheses. Age is presented as mean ± standard deviation, and other variables are listed as value counts. The train/validation and test sets utilized in our study are identical to those utilized in preliminary research [29]

At a 10-second interval, a total of 6410 video frames were collected from the intraoperative recordings, with 4661 and 1749 images for each set, respectively. 2307 (49.5%) images from the train-validation set and 635 (36.3%) images from the test set contained neural structure, i.e., spinal nerve roots, the dural sac surrounding the spinal cord, and the cauda equina. For these images, ground truth polygon masks were generated using LabelMe by a spine surgeon with more than four years of experience in BESS [25]. It should be noted that the labelled dataset is equivalent to that used in the previous study [15].

### Model overview

**Fig. 1:**
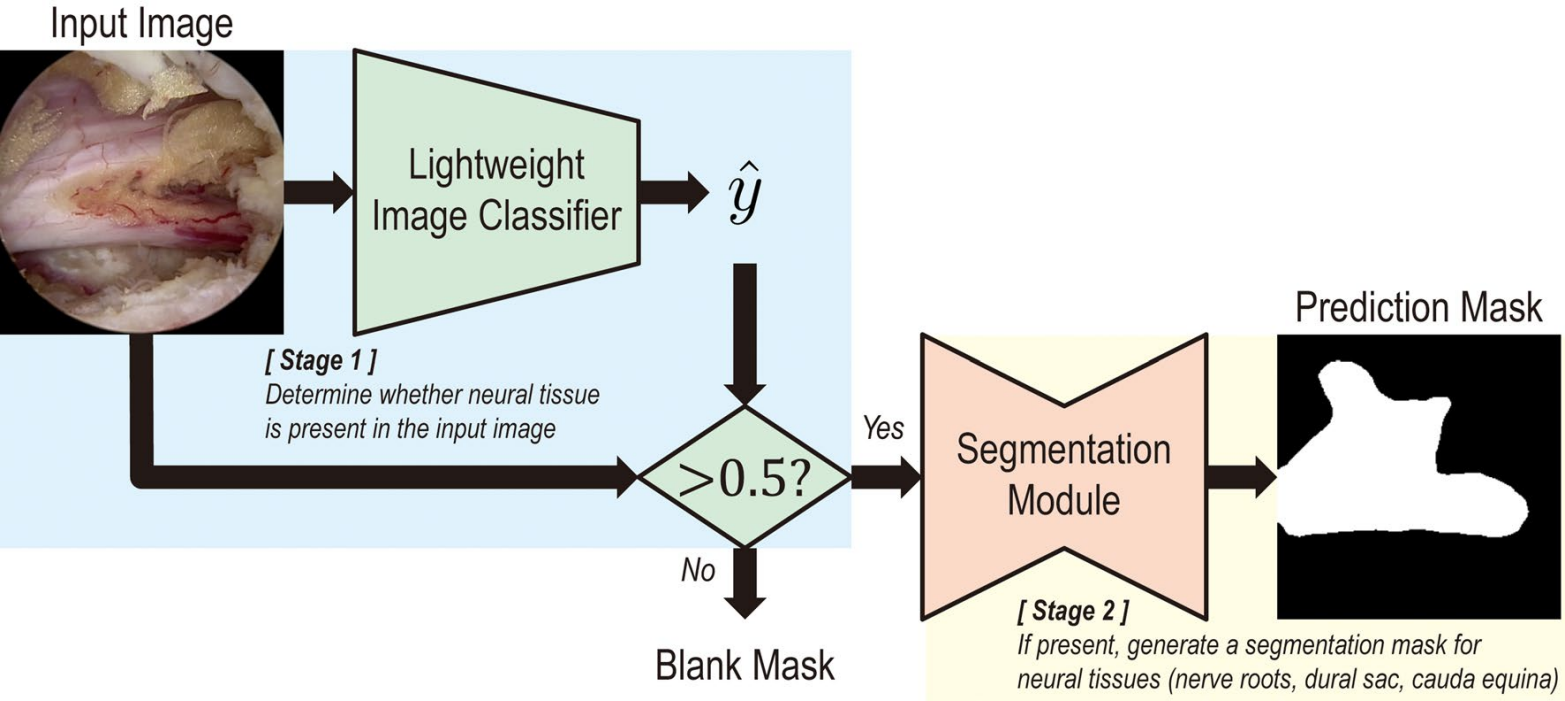
Overview of the proposed two-stage model. A two-stage model consisting of a classification and a segmentation module is illustrated. The first stage classifies whether neural tissue, i.e. nerve roots and the dural sac surrounding the spinal cord, and the cauda equina, are present in the input image. If present, the second stage identifies the neural tissue and generates a prediction mask

A two-stage module, illustrated in Fig. 1, is proposed to enhance the model’s robustness against false predictions. The first stage consists of an image classifier that indicates the presence of neural tissue in the input frame. To ensure fast inference speed, lightweight models, represented by ResNet18, ResNet34, and MobileNetV3-Large, were adopted [26, 27]. The second stage performs neural tissue segmentation, and six U-Net variant architectures, as well as SegFormer-B0 and SegFormer-B1, were trained [18–24]. High-performance models with state-of-the-art backbone architectures were not considered due to their computational demands, which often exceed the inference capabilities of contemporary hardware [28–30]. It should be noted that input resolution for the classifiers is 224 × 224, while that of U-Nets and SegFormers are 256 × 256 and 512 × 512, respectively.

### Image classification model

The image classifiers were trained for 50 epochs using three-fold cross-validation. Data augmentation, consisting of random rotation, zoom, and flip, was applied only during the training phase. Binary cross-entropy loss was used with the Adam optimizer and a learning rate scheduler that reduced the learning rate when validation loss failed to improve for a predefined number of epochs. For each architecture—ResNet18, ResNet34, and MobileNetV3Large—the set of hyperparameters that minimized the mean validation loss across the three folds was identified using random search with 10 trials. The area under the receiver operating characteristic curve (AUROC) on the test set was considered the primary outcome measure, while auxiliary metrics, including accuracy, sensitivity, specificity, precision, F1-score, false positive rate (FPR), and false negative rate (FNR), were also evaluated [31].

### Image segmentation model

#### U-net variant architectures

**Fig. 2:**
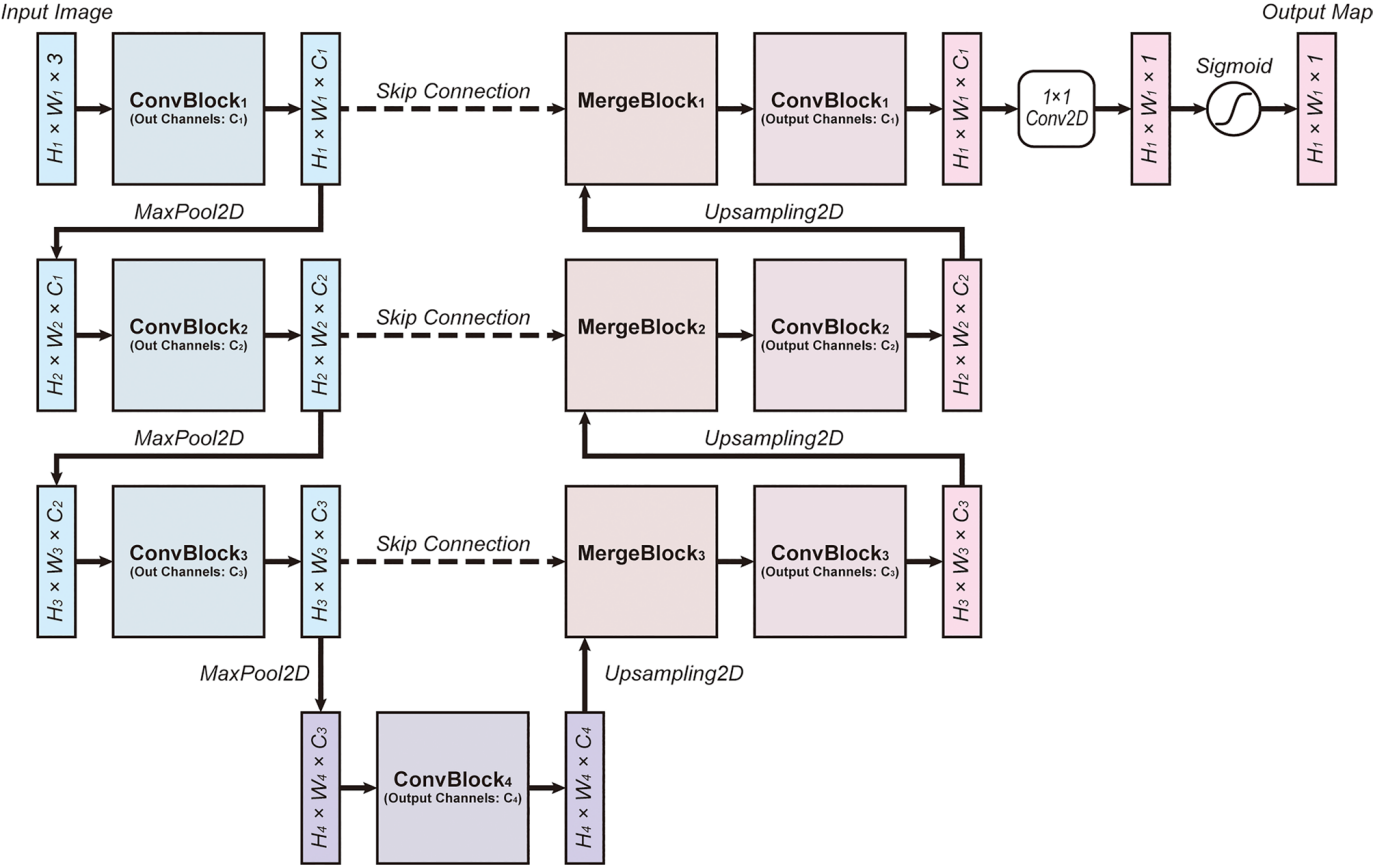
The base architecture of the deep learning model. The U-Net architecture was adopted for the present study [12]. H, W, and C refer to the height, width, and the number of channels of corresponding layers. ConvBlock consists of a stack of two 2D convolutional layers, and MergeBlock merges the data from the skip connection with the data upsampled from a layer of coarser resolution

Many studies have reported the effectiveness of U-Nets under resource-constrained environments, which is common in biomedical image segmentation tasks [19]. U-Net follows an encoder-decoder structure with skip connections directly linking pairs of distant layers with the same resolution, as illustrated in Fig. 2. The encoder is composed of a series of convolutional blocks (ConvBlock) and a maximum pooling layer (MaxPool2D), while the decoder contains merging units (MergeBlock) that merge skip connections and upsampled coarse features, followed by ConvBlocks. Finally, when the resolution of the original input is reached, the data is processed via a convolutional layer of kernel size 1 with sigmoid activation to generate the output map. In practice the width $\:{W}_{i}$, height $\:{H}_{i}$, and the number of channels $\:{C}_{i}$ at depth $\:i$ are set to satisfy the relations $\:{W}_{i+1}={W}_{i}/2$, $\:{H}_{i+1}={H}_{i+1}/2$, and $\:{C}_{i+1}=2{C}_{i}$.

**Fig. 3:**
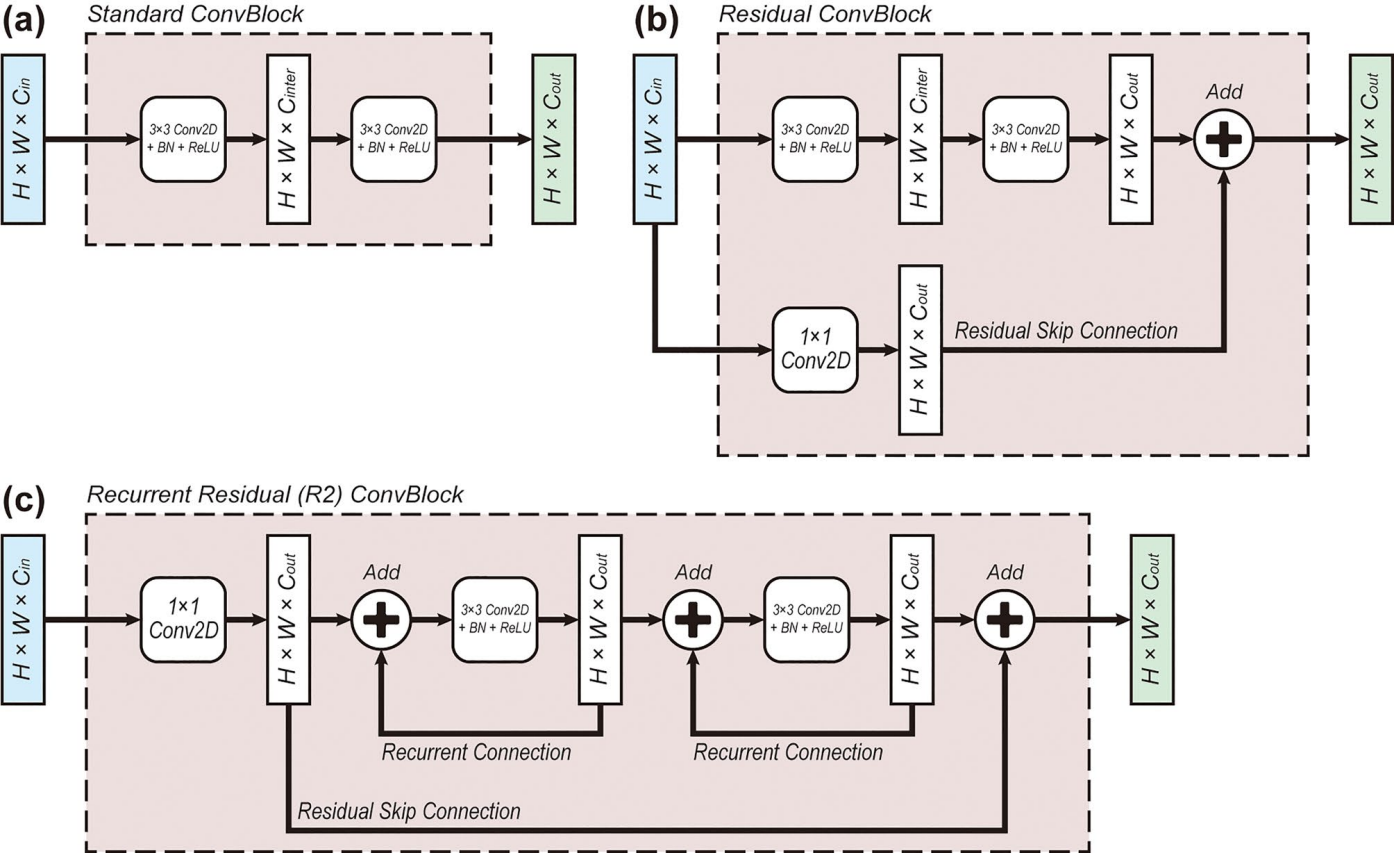
Two variations of the feature merging mechanisms (MergeBlocks). (**a**) Standard MergeBlock concatenates the data passed through the skip connection with the data upsampled from one level below. (**b**) Attention MergeBlock generates an attention map using the skipped and upsampled layers to allow the model to focus on important features [16]

As depicted in Fig. 3, we applied three types of variations to the ConvBlock. First, Fig. 3a shows the standard ConvBlock, which is used in the U-Net [18]. Figure 3b represents the residual ConvBlock (ResConvBlock), which introduces residual skip connections originally proposed in ResNet [21]. Theoretically, residual connections facilitate a more direct propagation of gradients throughout the training process and contribute to faster convergence and more stable learning even in deep neural networks with tens to hundreds of layers. Figure 3c is the recurrent residual ConvBlock (R2ConvBlock), in which recurrent connections are applied to each convolutional layer, facilitating the learning of deeper feature representations [20]. To elaborate, recurrent connections, when unfolded, can be thought of as a cascade of residual blocks sharing the same weights. Therefore, by exploiting redundancy, they can promote the learning of more complex feature representations with a slight increase in computational overhead.

**Fig. 4:**
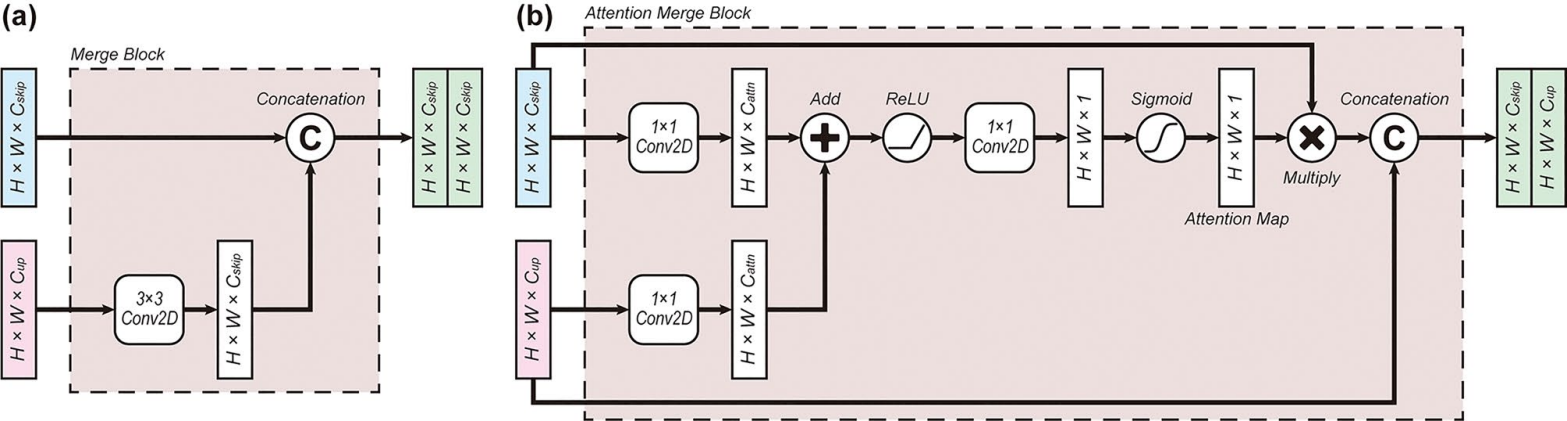
Three variations of convolutional blocks (ConvBlocks). (**a**) Standard ConvBlock is composed of two convolutional layers with batch normalization (BN) and rectified linear unit (ReLU) activation. (**b**) Residual ConvBlock includes a residual skip connection alongside the standard ConvBlock architecture [15]. (**c**) Recurrent residual (R2) ConvBlock adds recurrent connections to each of the convolutional layers and contains residual skip connections [14]

As illustrated in Fig. 4, two types of architecture for merging the skip connection and upsampled layer were proposed in our study. First, the standard MergeBlock in Fig. 4a concatenates the two inputs in a channel-wise manner, as implemented in [18]. The attention MergeBlock, adopted from Attention U-Net and depicted in Fig. 4b, incorporates attention mechanisms to allow the model to focus on more important features [22]. As shown in Fig. 3b, the attention mechanism introduces an attention map that works as a bottleneck layer, enforcing the learning of important features. It prevents features from becoming excessively sparse and allows the model to literally “attend” to relevant areas, thereby improving robustness even with limited data availability. With these building blocks, we designed six distinct architectures: U-Net, Residual U-Net (ResU-Net), Recurrent Residual U-Net (R2U-Net), Attention U-Net (AU-Net), Attention Residual U-Net (AResU-Net), and Attention Recurrent Residual U-Net (AR2U-Net). Their detailed characteristics, including input resolution and number of channels, are summarized in Table 2. It should be noted that R2U-Net and AR2U-Net were designed to contain only half the number of channels compared to other models to make the overall computational overload similar.

**Table 2:** Detailed characteristics of the architecture of U-Net variants used in the study

		U-Net	ResU-Net	R2U-Net	AU-Net	AResU-Net	AR2U-Net
Level 1	Resolution	(256, 256)	(256, 256)	(256, 256)	(256, 256)	(256, 256)	(256, 256)
	ConvBlock	Standard	Residual	R2	Standard	Residual	R2
	C_inter_	16	16	NA	16	16	NA
	C_out_	64	64	32	64	64	32
	MergeBlock	Standard	Standard	Standard	Attention	Attention	Attention
	C_attn_	NA	NA	NA	16	16	8
Level 2	Resolution	(128, 128)	(128, 128)	(128, 128)	(128, 128)	(128, 128)	(128, 128)
	ConvBlock	Standard	Residual	R2	Standard	Residual	R2
	C_inter_	32	32	NA	32	32	NA
	C_out_	128	128	64	128	128	64
	MergeBlock	Standard	Standard	Standard	Attention	Attention	Attention
	C_attn_	NA	NA	NA	32	32	16
Level 3	Resolution	(64, 64)	(64, 64)	(64, 64)	(64, 64)	(64, 64)	(64, 64)
	ConvBlock	Standard	Residual	R2	Standard	Residual	R2
	C_inter_	64	64	NA	64	64	NA
	C_out_	256	256	128	256	256	128
	MergeBlock	Standard	Standard	Standard	Attention	Attention	Attention
	C_attn_	NA	NA	NA	64	64	32
Level 4	Resolution	(32, 32)	(32, 32)	(32, 32)	(32, 32)	(32, 32)	(32, 32)
	ConvBlock	Standard	Residual	R2	Standard	Residual	R2
	C_inter_	128	128	NA	128	128	NA
	C_out_	512	512	256	512	512	256

Architectural components of the deep learning models, including input resolution and number of output channels, are listed. $\:{C}_{inter}$ and $\:{C}_{out}$ are the number of channels in the internal and output layers of the convolutional blocks, and $\:{C}_{attn}$ refers to the number of channels used for generating attention maps in the merging process. ResU-Net – Residual U-Net; R2U-Net – Recurrent Residual U-Net; AU-Net – Attention U-Net; AResU-Net – Attention Residual U-Net; AR2U-Net – Attention Recurrent Residual U-Net; R2 – Recurrent Residual; NA – not applicable

#### Segformer architecture

SegFormer achieves fast inference by combining a hierarchical transformer encoder, which extracts multi-scale features without relying on positional encoding, with a lightweight MLP decoder that efficiently fuses features from different encoder stages [24]. This architecture allows SegFormer to capture both local and global context while maintaining a simple and efficient design. By avoiding complex operations in the decoder and minimizing the number of parameters and computations throughout the model, SegFormer delivers both high segmentation accuracy and significantly faster inference compared to previous state-of-the-art methods. In our work, we adopted SegFormer-B0 and SegFormer-B1 models, which exhibit the least computational demands among the SegFormer family.

#### Model training

To promote the development of more robust and generalizable deep learning models, three-fold cross-validation was applied during the train/validation process. Dice loss on the train set and validation set was measured for each epoch to determine whether to apply early stopping and terminate the training procedure. Dice loss is defined in Eq. 1, where $\:{y}_{ij}\:$and $\:{\widehat{y}}_{ij}$ refer to the ground truth and predicted pixel value at $\:i$-th row and $\:j$-th column of the image, respectively, and $\:ϵ$ is a small value that prevents divergence [32, 33]. During the training phase, the data was expanded three-fold and was augmented with random rotation and flipping, while no other pixel-wise modifications were applied to the images. For each architecture, the set of hyperparameters resulting in the largest mean Dice similarity coefficient (DSC) across the validation set was determined using random search from a search space commonly accepted in practice.

1 $$\:Dice\:Loss=\:1-\frac{2\times\:{\sum\:}_{i,j}{y}_{ij}{\widehat{y}}_{ij}}{\left({\sum\:}_{i,\:j}{y}_{ij}+{\widehat{y}}_{ij}\right)+\varepsilon}$$

The training and testing processes were carried out using TensorFlow (version 2.13.0), PyTorch (version 2.6.0), and Scikit-learn (version 1.4.2) running inside a Python 3.11.9 environment on a computer with an Intel(R) Core(TM) i9-14900KF CPU, an NVIDIA GeForce RTX 4090 24GB graphics card, and 64GB of DDR5 RAM.

#### Performance assessment

DSC was our primary outcome measure, while metrics such as intersection over union (IoU), precision, recall, FPR, and FNR, and area under the precision-recall curve (AUPRC) were measured [34]. The confidence intervals of the performance metrics were obtained through bootstrap resampling of the test set, and statistical testing for model comparison was conducted likewise. P-values below 0.05 were considered statistically significant. The number of trainable parameters and floating point operations (FLOPs), as well as inference speed on our hardware, were measured to assess the real-time applicability of the proposed models. For qualitative evaluation, several randomly selected samples from the test set were visualized along with the prediction masks generated by each model.

## Results

### Image classification model

**Fig. 5:**
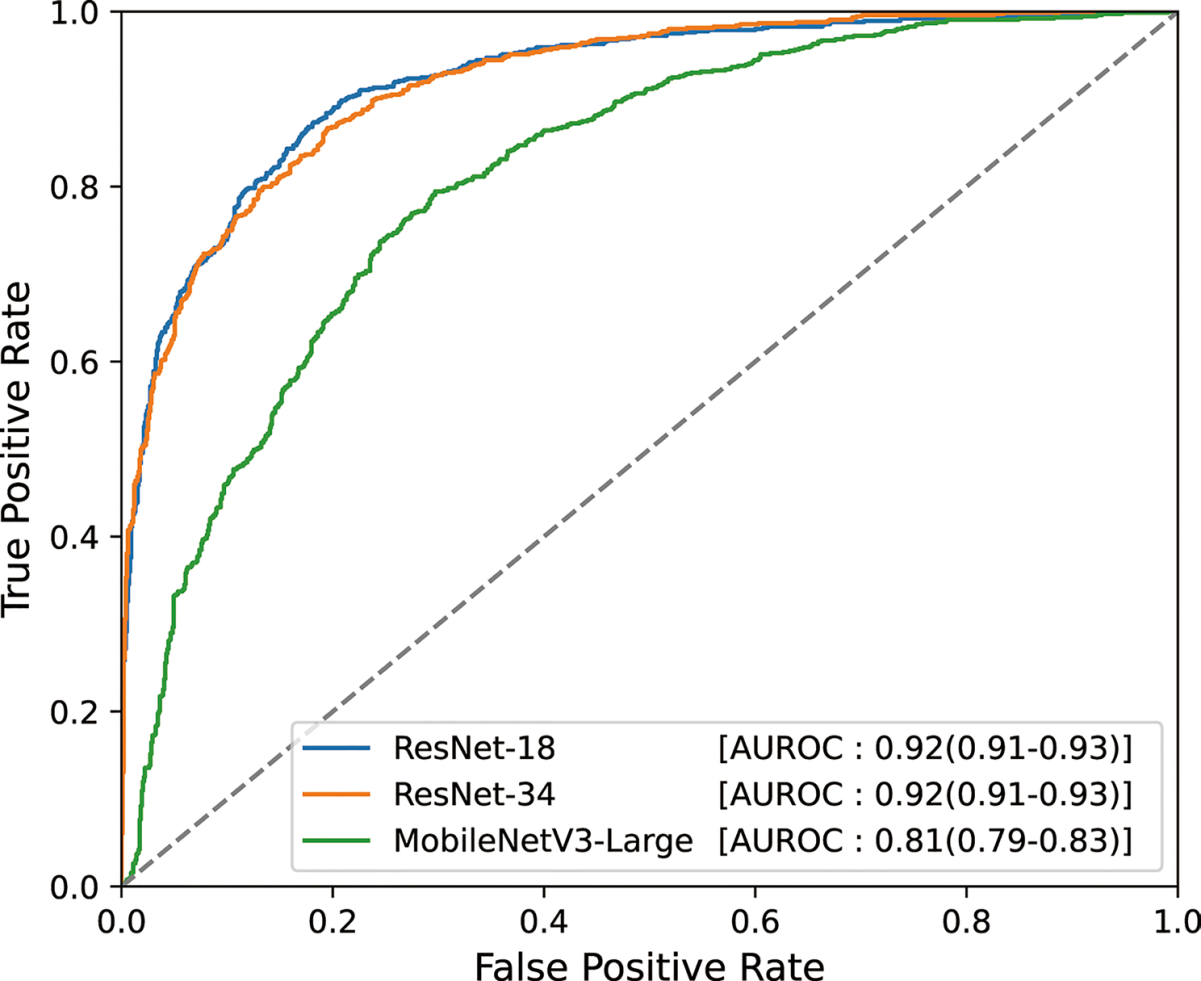
ROC curves on the test set for each classification model architecture. The receiver-operating characteristic (ROC) curves evaluated on the test set are plotted with AUROC scores. Values in parentheses indicate 95% confidence intervals estimated with bootstrap resampling. AUROC – Area under the receiver-operating characteristic curve

The performance of the classification models on the test set is summarized in Table 3; Fig. 5. The ResNet-18 model achieved the highest performance, with an AUROC of 0.92 (95% confidence interval [CI]: 0.91–0.93), accuracy of 0.84 (95% CI: 0.82–0.86), and F1-score of 0.81 (95% CI: 0.79–0.83). No statistically significant difference in AUROC was observed between ResNet-18 and ResNet-34 (*p* = 0.80), and none of the other metrics showed a significant difference between the two models. In contrast, the MobileNetV3-Large model performed worse than both ResNet-18 and ResNet-34 across all evaluation metrics (*p* < 0.001).

**Table 3:** Classification model performance on the test set

Architecture	AUROC	Accuracy	Sensitivity	Specificity	Precision	F1-score	FPR	FNR	NPV
ResNet-18	0.92 (0.91–0.93)	0.84 (0.82–0.86)	0.81 (0.78–0.84)	0.86 (0.84–0.88)	0.82 (0.79–0.85)	0.81 (0.79–0.83)	0.14 (0.12–0.16)	0.19 (0.16–0.22)	0.86 (0.83–0.88)
ResNet-34	0.92 (0.91–0.93)	0.83 (0.82–0.85)	0.77 (0.74–0.80)	0.88 (0.86–0.90)	0.83 (0.81–0.86)	0.80 (0.78–0.82)	0.12 (0.10–0.14)	0.23 (0.20–0.26)	0.83 (0.81–0.86)
MobileNetV3-L	0.81 (0.79–0.83)	0.74 (0.72–0.76)	0.73 (0.69–0.76)	0.76 (0.73–0.78)	0.70 (0.67–0.73)	0.71 (0.69–0.74)	0.24 (0.22–0.27)	0.27 (0.24–0.31)	0.78 (0.76–0.81)

The performance metrics of the image calssification models on the test set for each architecture are summarized. Values in parentheses indicate 95% confidence intervals. AUROC – area under the receiver-operating characteristic curve; FPR – false positive rate; FNR – false negative rate; NPV – negative predictive value

### Image segmentation model

**Fig. 6:**
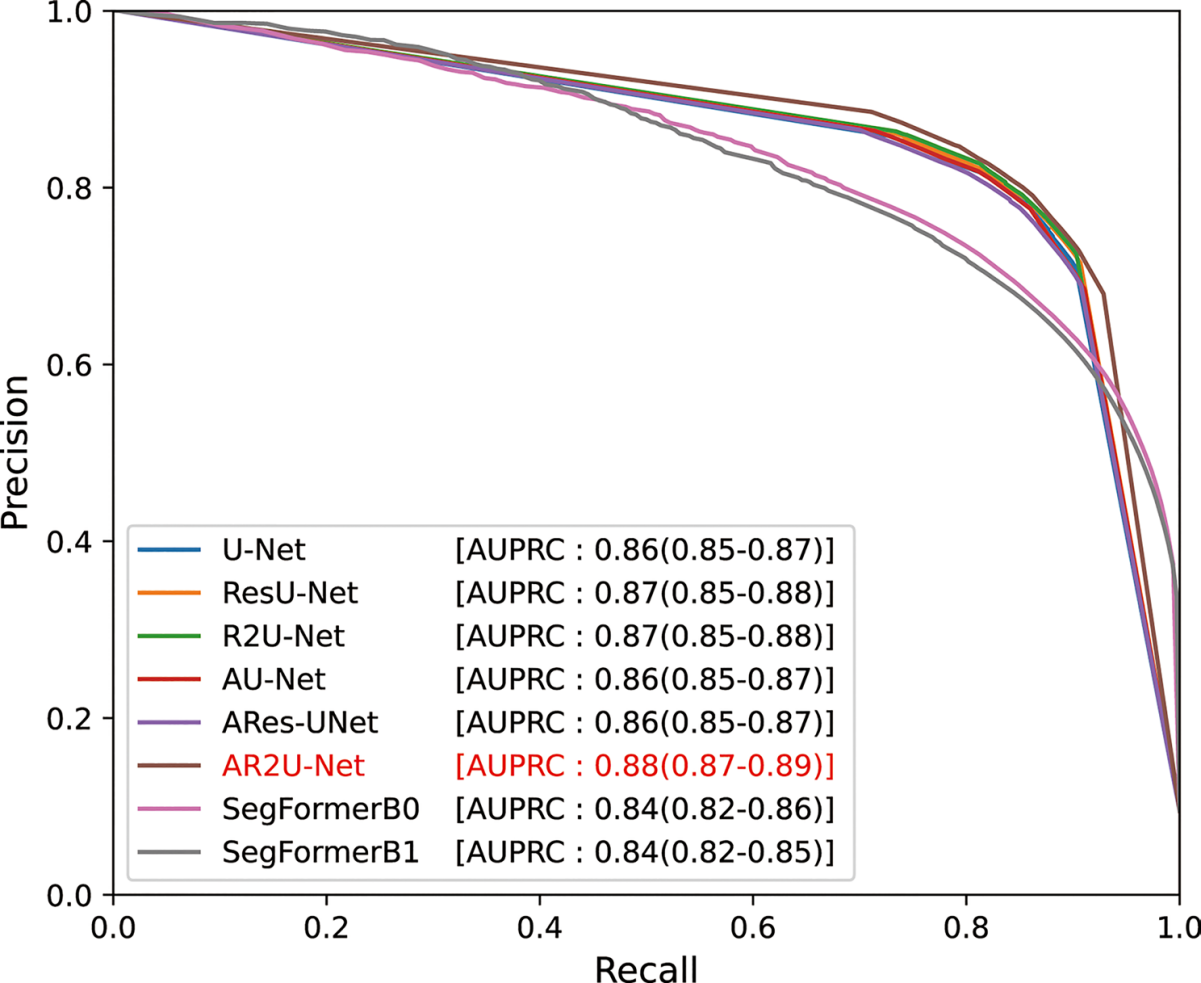
Precision-recall curves on the test set for each segmentation model architecture. The precision-recall curves obtained from the test set are plotted with AUPRC scores. Values in parentheses indicate 95% confidence intervals estimated with bootstrap resampling. AUPRC – Area under the precision recall curve; ResU-Net – Residual U-Net; R2U-Net – Recurrent Residual U-Net; AU-Net – Attention U-Net; AResU-Net – Attention Residual U-Net; AR2U-Net – Attention Recurrent Residual U-Net

The results of the segmentation models measured on the test set are presented in Table 4; Fig. 6. U-Nets generally demonstrated strong performance, with DSC of 0.79 (95% CI: 0.78–0.81) and IoU of 0.69 (95% CI: 0.67–0.70) for the base U-Net. Among them, AR2U-Net, which incorporates attention mechanisms and recurrent residual convolutions, achieved the highest AUPRC at 0.88 (95% CI: 0.87–0.89). It was significantly greater than those of the other U-Net variants (*p* < 0.001). In terms of DSC and IoU, AR2U-Net showed marginally better results, though the differences were not statistically significant.

**Table 4:** Segmentation model performance on the test set

Architecture	DSC	IoU	Accuracy	Sensitivity	Specificity	Precision	FPR	FNR	AUPRC
U-Net	0.79 (0.78–0.81)	0.69 (0.67–0.70)	0.94(0.93–0.94)	0.84 (0.82–0.85)	0.95 (0.95–0.96)	0.80 (0.78–0.81)	0.05 (0.04–0.05)	0.17 (0.15–0.18)	0.86 (0.85–0.87)
ResU-Net	0.80 (0.78–0.81)	0.69 (0.68–0.71)	0.94(0.94–0.94)	0.84 (0.82–0.85)	0.96 (0.95–0.96)	0.80 (0.79–0.82)	0.04 (0.04–0.05)	0.17 (0.15–0.19)	0.87 (0.85–0.88)
R2U-Net	0.80 (0.78–0.81)	0.69 (0.68–0.71)	0.94(0.93–0.94)	0.83 (0.82–0.85)	0.96 (0.95–0.96)	0.81 (0.79–0.82)	0.04 (0.04–0.05)	0.17 (0.15–0.19)	0.87 (0.85–0.88)
AU-Net	0.79 (0.78–0.81)	0.69 (0.67–0.70)	0.94(0.93–0.94)	0.84 (0.82–0.85)	0.95 (0.95–0.96)	0.80 (0.78–0.81)	0.05 (0.04–0.05)	0.17 (0.15–0.18)	0.86 (0.85–0.87)
AResU-Net	0.79 (0.77–0.80)	0.68 (0.66–0.70)	0.94(0.93–0.94)	0.83 (0.81–0.85)	0.95 (0.95–0.96)	0.79 (0.78–0.81)	0.05 (0.04–0.05)	0.17 (0.16–0.19)	0.86 (0.85–0.87)
AR2U-Net	0.80 (0.78–0.81)	0.70 (0.68–0.72)	0.94(0.94–0.94)	0.84 (0.82–0.85)	0.96 (0.95–0.96)	0.81 (0.80–0.83)	0.04 (0.04–0.05)	0.17 (0.15–0.19)	0.88 (0.87–0.89)
SF-B0	0.72 (0.71–0.74)	0.61 (0.60–0.63)	0.92(0.92–0.93)	0.72 (0.71–0.74)	0.96 (0.96–0.96)	0.78 (0.76–0.80)	0.04 (0.04–0.04)	0.28 (0.26–0.30)	0.84 (0.82–0.86)
SF-B1	0.70 (0.68–0.72)	0.59 (0.57–0.61)	0.92(0.92–0.93)	0.70 (0.68–0.72)	0.96 (0.95–0.96)	0.78 (0.76–0.80)	0.04 (0.04–0.05)	0.31 (0.29–0.33)	0.84 (0.82–0.85)

The performance metrics of the image segmentation models on the test set for each architecture are summarized. Values in parentheses indicate 95% confidence intervals. DSC – Dice Sorensen coefficient; IoU – intersection over union; FPR – false positive rate; FNR – false negative rate; AUPRC – area under the precision-recall curve; ResU-Net – Residual U-Net; R2U-Net – Recurrent Residual U-Net; AU-Net – Attention U-Net; AResU-Net – Attention Residual U-Net; AR2U-Net – Attention Recurrent Residual U-Net; SF – SegFormer

SegFormer-B0 achieved a DSC of 0.72 (95% CI: 0.71–0.74), IoU of 0.60 (95% CI: 0.59–0.61), and AUPRC of 0.84 (95% CI: 0.82–0.86) on the test set. While the differences were modest, SegFormer-B0 recorded significantly higher DSC and IoU compared to the more complex SegFormer-B1 model (*p* = 0.001), while no significant difference in AUPRC was observed between the two models (*p* = 0.55). When compared to the U-Net variants, the SegFormer models were significantly inferior to the U-Net models across all evaluation metrics (*p* < 0.001).

### Computational load analysis

Table 5 summarizes the computational demands of the deep learning models, including the number of trainable parameters, FLOPs, and inference speed. ResNet-based models generally had more trainable parameters than the segmentation models due to their final fully connected layers, but achieved fast inference times of 1–2 ms per frame. Given their strong performance and ability to filter out frames without neural tissue, this delay is acceptable. In addition, when considering the performance summarized in Table 3, ResNet-18 was identified as the most suitable architecture among the three candidates for the first stage.

**Table 5:** List of computational burden for each architecture

Architecture	Parameter Count (millions)	FLOPs (billions)	Inference time (ms)	Frame rate (FPS)
ResNet-18	11.2	3.6	1.2	830
ResNet-34	21.3	7.3	1.6	610
MobileNetV3-L	4.2	0.4	2.1	470
U-Net	3.3	46.6	27	37
ResU-Net	3.7	50.7	35	29
R2U-Net	6.4	59.1	43	23
AU-Net	2.0	22.5	25	40
AResU-Net	2.5	28.1	33	30
AR2U-Net	2.7	45.7	41	24
SegFormer-B0	3.7	2.7	14	72
SegFormer-B1	13.7	5.2	15	67

The number of parameters, floating point operations (FLOPs), inference time on our hardware, and frame rate are listed. FLOPs – floating point operations; FPS – frames per second; ResU-Net – Residual U-Net; R2U-Net – Recurrent Residual U-Net; AU-Net – Attention U-Net; AResU-Net – Attention Residual U-Net; AR2U-Net – Attention Recurrent Residual U-Net

The segmentation models achieved processing speeds suitable for real-time application, operating at 25–40 frames per second (FPS) for U-Net variants and approximately 70 FPS for SegFormer-B0 and SegFormer-B1. Considering both segmentation performance and inference speed, U-Net and AU-Net were identified as the most practical choices. When combined with the ResNet-18-based classifier and applied to a video stream of 30 FPS and resolution of 256 by 256, the proposed pipelines worked smoothly without delay.

### Qualitative analysis

**Fig. 7:**
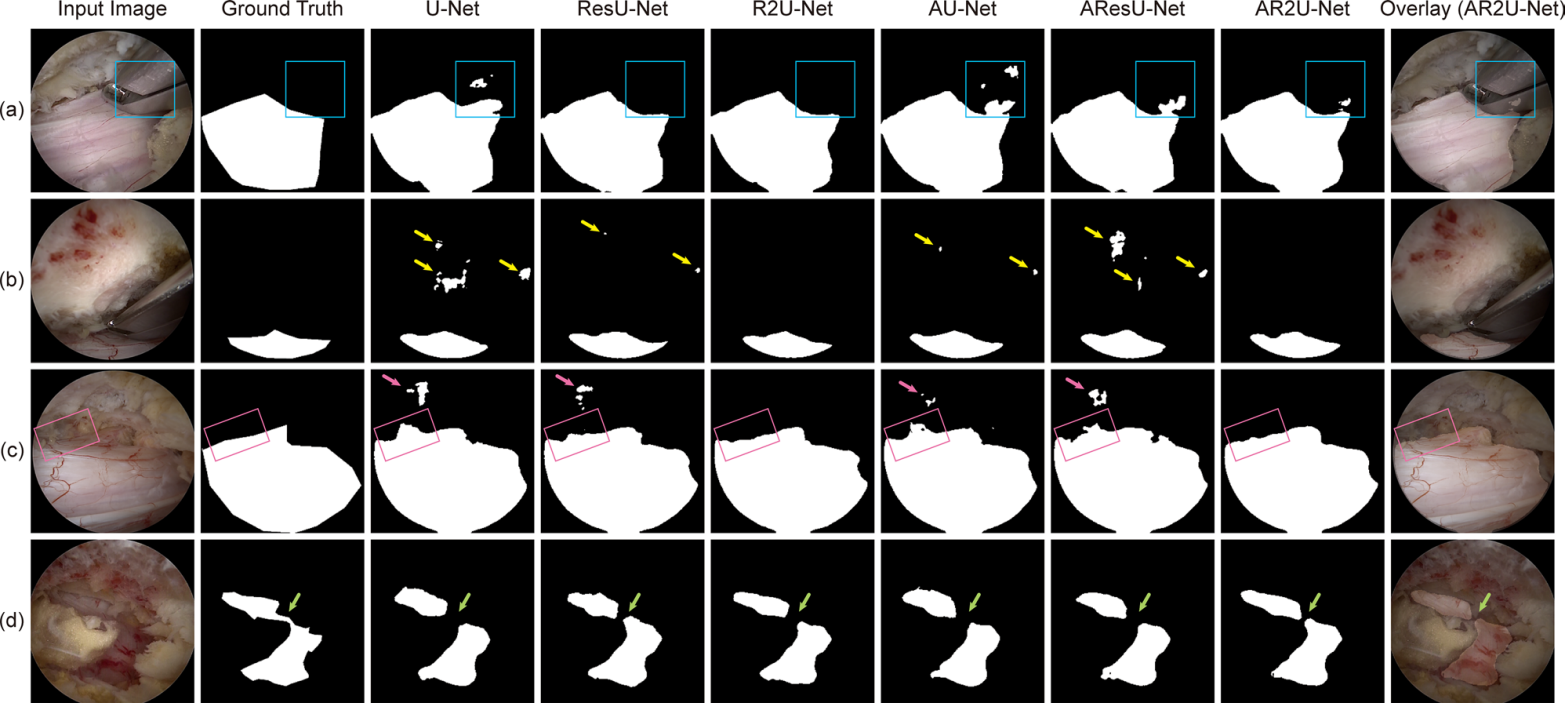
Test samples illustrating the difference among U-Net variant architectures. The input image, ground truth segmentation label, and prediction masks generated from the six U-Net variant architectures are shown. (**a**) The architectures produce different outputs for neural tissue reflections on a surgical instrument [blue rectangle]. (**b**) False positive artifacts [yellow arrows] are effectively suppressed in architectures with recurrent residual convolutional blocks. (**c**) Models exhibit varying responses to false positive regions [pink arrows], with nerve root boundaries being more accurately delineated in models incorporating recurrent residual blocks [pink boxes]. (**d**) Different segmentation outputs are observed for fine structural details [green arrows]. (**e**) False negative regions [orange boxes] differ across architectures. ResU-Net – Residual U-Net; R2U-Net – Recurrent Residual U-Net; AU-Net – Attention U-Net; AResU-Net – Attention Residual U-Net; AR2U-Net – Attention Recurrent Residual U-Net

Several samples were randomly selected from the test set for qualitative comparison among models. Figure 7 lists a few cases that effectively illustrate the difference among U-Net variants. The models were capable of identifying visible neural tissues, regardless of architectural differences. Performance differed most prominently at tissue boundaries, where more complex models generally demonstrated better delineation accuracy. For instance, in Fig. 7a, complex models successfully suppressed the areas in which neural tissue was reflected from surgical instruments, while in Fig. 7c, R2U-Net and AR2U-Net accurately segmented nerve roots partially obscured by shadows. Although these differences may not be strongly reflected in gross performance metrics such as DSC or IoU, they are expected to have meaningful implications for the quality of model outputs in clinical settings.

**Fig. 8:**
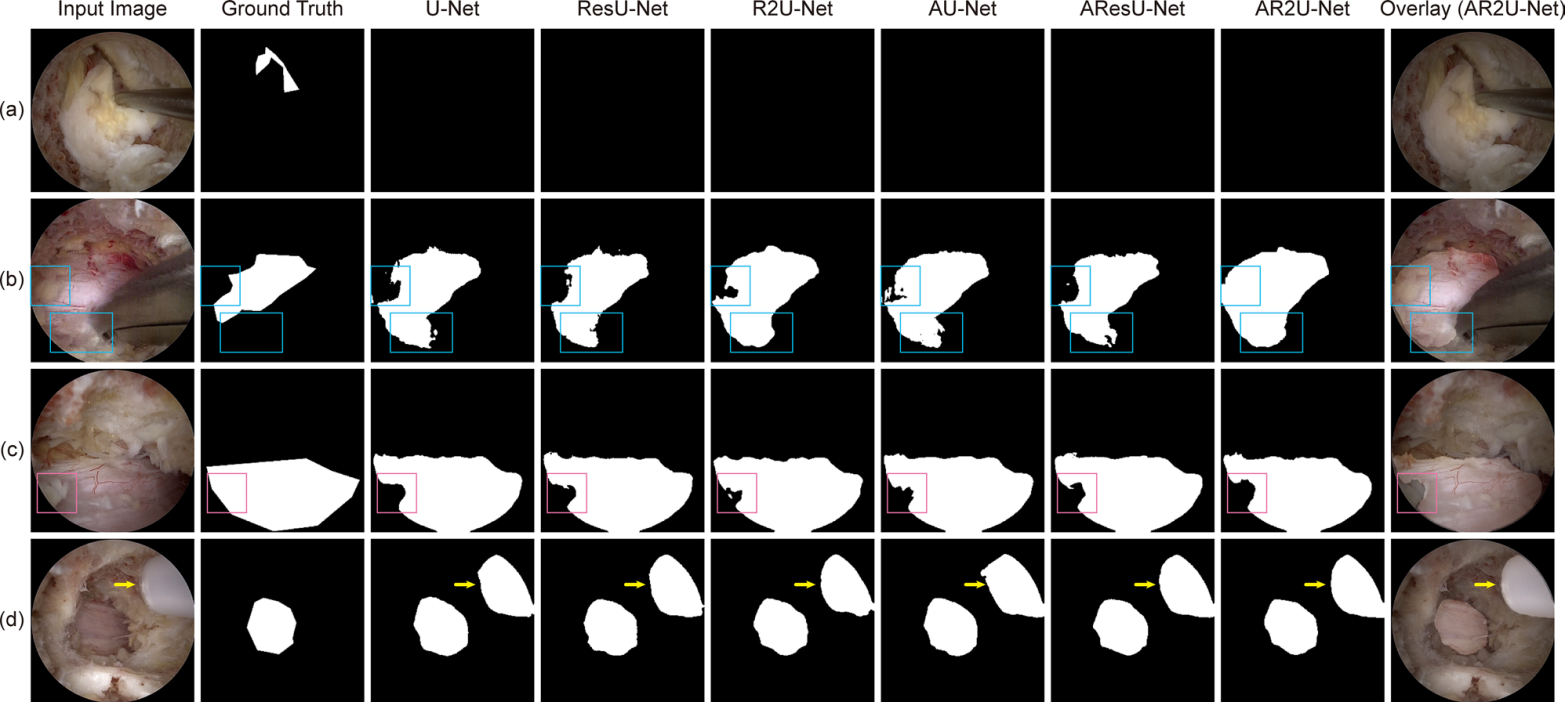
Challenging and failing test samples. The input image, ground truth segmentation label, and prediction masks generated from the six U-Net variant architectures are shown. (**a**) None of the models successfully segment neural tissue with small and irregular shapes. (**b**) The models produce varying outputs for neural tissue covered by perineural fat [blue boxes]. (**c**) Regions with prominent perineural fat tend to be excluded across models [pink boxes]. (**d**) The models fail to exclude a surgical instrument [yellow arrows]. ResU-Net – Residual U-Net; R2U-Net – Recurrent Residual U-Net; AU-Net – Attention U-Net; AResU-Net – Attention Residual U-Net; AR2U-Net – Attention Recurrent Residual U-Net

There existed some cases, as listed in Fig. 8, where the models failed to provide accurate predictions. As shown in Fig. 8a, neural tissue was not recognized in frames where the field of view was severely limited, while in Fig. 8b and c, partially occluded regions by fat tissue led to inconsistent predictions among the models. In addition, as visualized in Fig. 8d, a rare instance was observed where a tubular-shaped instrument was mistakenly identified as neural tissue.

## Discussion

Remarkable advancements in deep learning within the field of computer vision have enabled automated image classification and object detection, approaching or even surpassing human-level performance in some cases [26, 29, 35]. In particular, the U-Net family has demonstrated outstanding performance in biomedical image segmentation tasks, while vision transformers have emerged as another group of reliable architectures [18, 19, 36, 37]. Although deep learning has been increasingly applied to intraoperative videos, its use in BESS remains in the early stages. This section discusses the novelty and contributions of the present work, followed by its limitations and potential strategies for improvement.

### Comparison with preliminary studies

We build upon the previous research conducted by Lee et al., which proposed a U-Net model for the segmentation of nerve roots and the spinal cord, reporting DSC of 0.824, IoU of 0.701, and AUPRC of 0.890 on the test set [15]. First, we developed an image classifier module that can screen whether the input image contains neural tissue, thereby enhancing robustness against false predictions. In addition, diverse models were trained, including U-Nets augmented with recurrent residual convolution and attention mechanisms, as well as transformer-based models represented by SegFormer. Lastly, computational demands were rigorously analyzed to assess the feasibility of real-time implementation.

One of the key findings of this study is that the addition of a lightweight image classifier, operating at a fast speed of less than 2 ms per image, can be placed in front of a heavier segmentation model to reduce false predictions. This two-stage approach is not limited to neural tissue detection but may be generalized to other tasks as well. For instance, Cho et al. developed a deep learning-based module for detecting surgical instrument tips using a dataset of 2310 BESS video frames, all containing surgical instruments [16]. As their model may be vulnerable to false predictions in frames without surgical instruments, our strategy could be readily applied to enhance robustness.

### Comparison of model architectures

ResNet significantly outperformed MobileNetV3-Large in the image classification task. Although MobileNet aims to improve computational efficiency through the use of inverted residual blocks, its substantially lower parameter count may have limited its performance in this specific task [27]. As no statistically significant performance difference was observed between ResNet-18 and ResNet-34, the former was considered the most appropriate choice for this task due to its lighter architecture.

Although the AUPRC of AR2U-Net was statistically significantly higher than those of other U-Net variants, the improvement was not as pronounced as theoretically expected. Thus, the benefits of residual connections, recurrent convolutions, and attention mechanisms were found to be minimal. On the contrary, these components increased model complexity, resulting in longer inference times and potentially hindering real-time applicability. Accordingly, U-Net or AU-Net, both of which can operate at speeds exceeding 30 FPS, are considered more suitable for practical use. In the future, as hardware capabilities continue to improve, more complex models with higher performance may be adopted, and higher input resolutions may also become feasible.

A noteworthy finding is the substantially inferior performance of SegFormers compared to U-Nets. This is presumed to be attributable to the “data-hungry” nature of vision transformers, which typically require significantly larger datasets for effective training [38, 39]. Unlike convolutional neural networks that build up local receptive fields in a hierarchical manner by leveraging translational invariance, transformers heavily rely on attention mechanisms to model global relationships among pixels and patches [40–42]. The latter approach is generally more complex and thus demands more data. Consequently, the full potential of the transformer architecture may not have been realized in our setting, where the dataset size was limited to a few thousand samples.

### Limitations and future directions

#### Generalizability

Generalizability should be critically considered when developing deep learning models for clinical purposes [43]. In the present study, the dataset was split at the patient level to prevent potential memoization during testing. In addition, video frames were extracted from throughout the entire BESS procedure to promote heterogeneity of the inputs. Nevertheless, it must be acknowledged that the absolute number of surgical videos (29 cases) is relatively small, inevitably resulting in some degree of redundancy within the dataset. Given the data-hungry nature suspected during the training of transformer-based models, further expansion of the dataset will be necessary in future work.

The most desirable approach to enhance generalizability would be to construct a dataset using multiple operators across multiple institutions [43–45]. Although this was not feasible in the present study due to the early stage of deep learning applications in BESS and time constraints, external validation using datasets from other institutions is currently being planned through inter-institutional collaboration. Furthermore, a temporal validation study is also under consideration, in which surgical cases are selected to avoid overlapping time intervals, thereby enabling an assessment of robustness against variations in surgical techniques [46].

Although the ground truth labels were annotated by a spine specialist with years of BESS experience, the use of a single annotator inevitably carries the risk of personal bias. Given that neural tissue is generally well distinguishable to the naked eye, the current labels can still be considered sufficiently reliable. However, involving multiple expert clinicians in the annotation process would likely lead to a higher-quality dataset by reducing individual subjectivity and enhancing label consistency.

#### Spatiotemporal context-awareness

In biomedical imaging AI, achieving high performance alone is insufficient; models must be capable of recognizing contextual cues and reasoning in a manner that closely resembles human cognition [47]. In this study, predictions were made on a frame-by-frame basis, without incorporating either temporal or anatomical information despite their importance in actual surgical settings. Temporal continuity provides valuable context before and after specific events, particularly in situations involving transient visual occlusions caused by bleeding or bubbles, where single-frame models may lack robustness. Similarly, anatomical structure plays a critical role in understanding the spatial relationships between neural tissue and surrounding elements such as fat, bone, and surgical instruments. Ignoring these structural cues may lead to biologically implausible predictions, such as disjointed or fragmented segmentation masks.

To capture temporal information in videos, recent studies have proposed strategies that combine convolutional neural networks (CNNs) with sequential architectures [48]. Moreover, transformer-inspired models have garnered increasing attention as a core methodology for integrating image and time-series data in a multimodal fashion, leading to the development of strategies that actively leverage their flexibility and representational power [49, 50]. While such methods inevitably increase model complexity and computational cost, they offer a promising path toward more context-aware and clinically applicable solutions.

The cases presented in Fig. 7 illustrate that augmenting segmentation models with various architectural components can improve tissue delineation and reduce false predictions. However, such enhancements often require substantial computational overhead, raising concerns about their real-time applicability. One potential solution is to adopt a multi-label strategy, as proposed by Bu et al., by annotating not only neural tissue but also surrounding anatomical structures and surgical instruments, thereby enabling the model to learn contextual relationships among different entities [17]. Structural awareness can also be improved through loss function design. For instance, incorporating boundary-aware constraints such as boundary loss or Hausdorff distance loss into standard Dice loss may enhance tissue delineation [33]. Furthermore, employing higher-level perceptual losses, such as structural similarity loss or perceptual loss widely used in GANs, could help guide the model toward outputs more closely aligned with human perception [51–53].

#### Clinical applicability

This study primarily aimed to assess the feasibility of applying deep learning to real-time BESS videos. However, our ultimate goal is to translate this technology into the operating room and generate tangible clinical value. As a next step, a usability study could be conducted in a virtual environment to evaluate the user experience and learning curve for novice surgeons without risking patient safety—a strategy increasingly adopted in robotic and laparoscopic surgery research [54–56]. Eventually, a prospective clinical trial could be designed to assess whether deep learning–based visual augmentation can reduce the incidence of nerve injury and demonstrate non-inferiority compared to conventional surgical techniques.

## Conclusions

As discussed earlier, deep learning applications for spinal endoscopy has not been well established despite the their clinical significance. In the present work, we addressed the limitations of preliminary research by introducing a fault-tolerant two-stage framework, promoting model diversity, and conducting rigorous analyses on computational efficiency. The resulting model was executable in real-time 30 FPS video stream within modern hardware configurations, implying that the proposed methodology can be readily applied for intraoperative visual augmentation, though sufficient evidence should be collected through usability studies and clinical trials.

## Data Availability

The datasets generated and/or analyzed during the current study are not publicly available due to privacy concerns, ethical considerations, and legal restrictions protecting patient confidentiality but are available from the corresponding author on reasonable request.

## Abbreviations

AI: Artificial intelligence
AR2U-Net: Attention recurrent residual U-Net
AResU-Net: Attention residual U-Net
AU-Net: Attention U-Net
AUPRC: Area under the precision-recall curve
AUROC: Area under the receiver-operating characteristic curve
BESS: Biportal endoscopic spine surgery
CI: Confidence interval
DSC: Dice similarity coefficient
FLOP: Floating point operations
FNR: False negative rate
FPR: False positive rate
FPS: Frames per second
IoU: Intersection over union (Jaccard score)
R2U-Net: Recurrent residual U-Net
ResU-Net: Residual U-Net
